# Friction Characteristics Analysis of Rubber Bushing with a Bionic Flexible Contact Surface Based on the Convex Hull Structure

**DOI:** 10.3390/polym15030606

**Published:** 2023-01-24

**Authors:** Ce Liang, Min Li, Yi Li, Jicai Liang, Qigang Han

**Affiliations:** 1 Key Laboratory of Automobile Materials, Ministry of Education, College of Materials Science and Engineering, Jilin University, Changchun 130025, China; liangce@jlu.edu.cn (C.L.); limin20@mails.jlu.edu.cn (M.L.); henrylee@jlu.edu.cn (Y.L.); liangjc@jlu.edu.cn (J.L.); 2 Chongqing Research Institute, Jilin University, Chongqing 401123, China; 3 Roll Forging Research Institute, Jilin University, Changchun 130025, China

**Keywords:** bionic tribology, rubber bushing, finite element analysis, flexible surface

## Abstract

Inspired by the convex hull structure of the dung beetle head’s surface, we extracted the non-smooth surface morphology of its head and designed a rubber bushing with a representative structure according to the bionics principle. According to the fitting results of the test data, Ogden N3-Prony N3 was selected as the hyper-viscoelastic constitutive model of the rubber material. Then, the two-direction (radial, axial) motion characteristics of the flexible friction pair in the rubber bushing were systematically analyzed from the aspects of stress, strain and thermal effect through the combination of numerical simulation and experimental research. Finally, the bionic design with the best drag reduction and wear resistance was determined.

## 1. Introduction

In recent years, people’s demands for vehicle performance have been upgraded from aiming to be less laborious and time-consuming to aiming for higher comfort and safety. The development of the automobile industry is now relatively mature, and technical advancements in the field of noise and vibration have significantly reduced interior noise in the cabin of a vehicle, which has, in turn, placed an emphasis on exterior noise. Rubber bushing is extensively used in automobile chassis for vibration attenuation and noise reduction owing to its merits, such as low cost and damping properties to dissipate energy [1]. As a connection between components, rubber bushing transfers movement from one part to another. In some cases, the bushing needs to withstand forces from multiple directions. When the car moves on a rough road, the bushing may be subjected to radial and other directional impact. When the car undergoes emergency braking or turning, the bushing is subjected to torsional and axial excitation. Hence, the working condition of a bushing is generally unpredictable [2]. In this case, the non-negligible contact friction between flexible friction pairs of bushing accelerates the wear [3]. In order to solve the problem of vibration and noise generated by serious wear and aging of rubber bushing, a kind of working surface designed with good friction and a good drag reduction effect is strongly needed.

Considering the waste of production time and material losses resulting from the experimental approaches, more systematic and analytical approaches are desirable [4]. From the perspective of rubber bushing to improve Noise-Vibration-Harshness (NVH) performance, the first and most in-depth type of research is to accurately calculate the dynamic stiffness of rubber bushing. For the sake of high efficiency and accuracy, researchers began to study the constitutive model of rubber material. Bagley and Torvik [5] successfully introduced the concept of fractional derivatives into the viscoelastic model of rubber bushings, which can describe the frequency dependence of rubber bushings with a small number of parameters. Joberg [6] proposed a multi-parameter generalized Maxwell model, which can accurately describe the dynamic characteristics of rubber bushing in a wide frequency domain. The model is still applied to the simulation analysis of large nonlinear finite element analysis software such as ABAQUS. Horiuchi [7] used several dynamic characteristics of rubber bushing under a vibration frequency up to 100 Hz to derive an equation which could calculate the dynamic stiffness at any amplitude only by identifying the dynamic stiffness and the loss factor, which greatly reduced the difficulty of calculating the dynamic stiffness. Rivas-Torres et al. [8] used a genetic algorithm to establish a mathematical model that could optimize structural parameters in a few minutes and compared it with the stiffness estimation results of the simulation model. He found that the performance of the two curves was highly consistent with the same target, and the error was less than 5%. With the help of these constitutive models and calculation methods, a series of achievements have been made in the structural design of rubber bushing. Kim et al. [9] proposed a geometric parametric design method and used nonlinear finite element software to simulate the performance of an engine mount. The analysis results verified the effectiveness of the method. Due to the limitation of the software version, the author only used a two-dimensional model to simulate the contact situation, and the final analysis result was quite different from the initial goal. Kaya et al. [10] used a differential evolution algorithm to optimize the shape of rubber bushing model whose constitutive model is a hyperelastic model, and the appropriate optimization design parameters were determined. However, the viscoelastic property of rubber material was not considered. When the deformation is large, the results will be inaccurate without considering the influence of viscoelasticity on the mechanical properties. Even for simpler static mechanical properties, the lack of viscoelasticity parameters may lead to results that deviate significantly from reality. Li et al. [11] optimized the geometrical parameters of rubber suspension components by combining a neural network with a genetic algorithm. The analysis results showed that the design method for optimizing the structural parameters of the rubber suspension element is reasonable. Kemna et al. [12] found that the torsional stiffness of the rubber bushing could be decreased by reducing the inner sleeve diameter and the thickness of the elastomer pad, resulting in a high performance suspension bushing with a radial to torsional stiffness ratio of 30:1. However, the above research on the design and optimization of the stiffness characteristics of rubber bushing cannot solve the problem of noise.

Abnormal noise is a result of a complex nonlinear phenomenon which is always caused by relative motion between two surfaces of a friction pair [13]. Kinkaid et al. [14], Ibrahim [15,16], Papinniemi et al. [17], Feeny et al. [18], and Akay [19] have comprehensively introduced the main theorems and mechanisms of noise and vibration; their respective analytical, numerical and experimental studies are also presented for the complex friction-induced phenomena. Nam et al. [20] studied the changes in friction coefficient and the characteristics of the contact surfaces for two lubricated surfaces using a reciprocating friction tester. The results show that friction noise is induced by the reduction of the lubricant causing the increase of the friction coefficient. Kang et al. [21] studied the influence of different factors on the occurrence of squeak noise and the relationship between friction force and noise through the friction experiments of rubber bushing, then squeak noise prediction models were constructed. However, studying the relationship between the change of friction force and abnormal noise is insufficient. It is more important to effectively reduce the friction coefficient on the surfaces of rubber bushing to reduce the probability of squeak noise.

Recently, researchers have paid more attention to bionic optimization methods [22]. Therefore, obtaining design inspiration from nature is a top priority in today’s research [23]. Gu et al. [24] established a rubber sealing model with bionic dimpled characteristics based on the theory of bionic dimpled drag reduction and the principle of rubber sealing. This design scheme solved the problem of sealing between the barrel and the rubber ring of the shell body during a launching process of an aerodynamic extinguishing cannon. Ma et al. [25] prepared semimetallic friction composites with and without a bionic non-smooth structure, then they tested to evaluate their friction wear properties. The results found that the bionic structure of testing samples had a better wear resistance and provided a stable friction coefficient. Wang et al. [26] prepared the bionic carp scale appearance pattern on a Ti6A14V surface by laser surface texturing technology. The results showed that the dry friction factor of the bionic carp scale morphology Ti6A14V reduced by 45% compared to those without bionic carp scale morphology. Jia et al. [27] designed anti-adhesion structures of press rollers based on earthworm movement characteristics. The results showed that the rubber bulge of the press roller had a good effect of soil adhesion reduction. Inspired by the surface structure of a giant cactus, with the maturity of bionic technology, bionic design was no longer limited to metal, and polymer materials, such as rubber, were also used. Mao et al. [28] introduced the bionic non-smooth structure of pits into tire tread compound preparation by referring to the bionics concept. Li Chuxi et al. [29] used 3D printing technology to print flexible material surfaces with circular concave, circular convex and square concave texture structures. The experimental results showed that the friction and drag reduction performance of the circular concave microtexture was the best.

A lot of research has been undertaken and many improvements have been achieved in the design of bionic structures and the optimization of the geometry of rubber bushing. However, no one has incorporated the bionic idea into the bushing design. Inspired by the fact that the non-smooth morphology of the dung beetle head’s surface can reduce soil resistance, the non-smooth surface with convex hulls was extracted and analyzed. According to the principle of bionics, a representative structure was designed on the surface of standard bushing. The basic test data of rubber materials were processed by the fitting function that comes with ABAQUS. According to the fitting effect, Ogden N3-Prony N3 was selected as the hyper-viscoelastic constitutive model of the rubber material. The research method adopted in this paper is a method combining numerical simulation and experiment. The two direction (radial, axial) mechanical properties of the flexible friction pair in the rubber bushing were systematically analyzed from the aspects of stress, strain and thermal effect. Finally, the bionic surface with the best drag reduction and wear resistance was identified to reduce the noise. The front axle rocker arm rubber bushing designed on the basis of bionics principles can improve the vehicle comfort and handling stability. At the same time, the design concept can also provide theoretical guidance for the development of rubber bushing in the future.

## 2. Materials and Methods

### 2.1. Bionic Design of the Rubber Bushings

**Figure 1:**
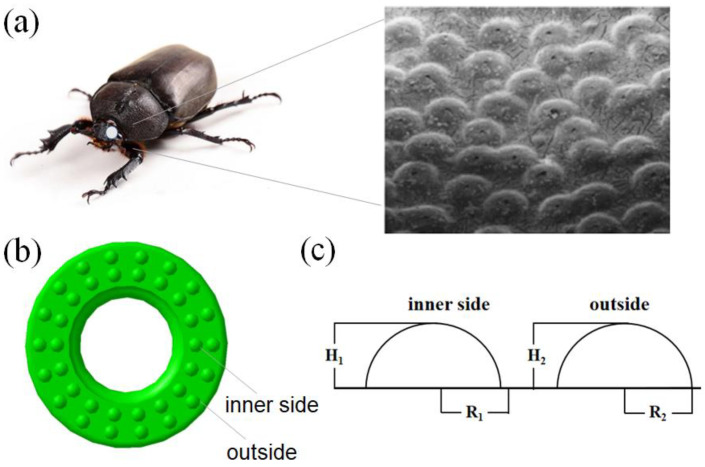
(**a**) Appearance of dung beetle, and the microscopic morphology of the dung beetle head’s surface (reprinted with permission from ref. [30]). (**b**) Convex hull distribution of bushing surface. (**c**) Dimensions of the convex hull section.

Convex hull geometry widely exists on the surface of soil cave animals. This structure of animals protects them by reducing drag and increasing surface wear resistance when they squeeze or rub against the soil. Inspired by the fact that the convex hull structure on the dung beetle head’s surface can reduce the resistance, the non-smooth surface of the rubber bushing was designed according to the principles of bionics. The microscopic morphology of the dung beetle head’s surface has been widely studied [30], and is shown in Figure 1, and the irregular convex hulls with a shape close to a semi-ellipse are distributed on the surface. Considering the irregular shape of the convex hull and the difficulty of processing the non-smooth shape, we abstracted the convex hulls into a simple spherical coronal solid structure. These coronal structures are evenly distributed on the surface of the bushing in two equidistant rings, as shown in Figure 1b. The dimensions of the convex hull are shown in Figure 1c, the radius of the inner convex hull is R_1_, the height is H_1_, the radius of the outer convex hull is R_2_ and the height is H_2_. The design idea of the corresponding dimensions is as follows. First, the convex hulls on the surface of a dung beetle’s head radiate outwards from a central point. After mathematical calculations, we found that the ratio of the height to the radius of the convex hull is about 0.4~1. The ratio of the height to the radius of the bionic convex hull size designed by us is also in the above range. Second, we considered the hardness of the rubber material, rubber bushing dimensions and preparation process, as well as the operating characteristics of bushing, and the dimensions of bionic units were set as mm. Third, in order to study the influence of convex hull size uniformity on surface characteristics, we made two design schemes with the same size and different size of the inner and outer convex hull. The specific dimensions are shown in Table 1.

### 2.2. Finite Element Modelling

**Figure 2:**
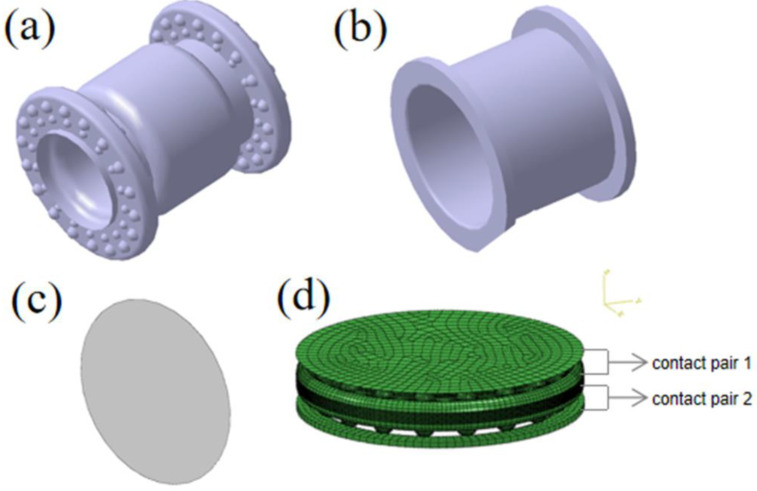
(**a**) Rubber body; (**b**) outer sleeve; (**c**) steel plate; (**d**) assembly.

The rubber bushing in this study belongs to the inner sleeve bonding and the outer sleeve pressing type bushing which was roughly cylindrical in shape. The metal part includes a metal inner sleeve, an inner skeleton and an outer sleeve, which are connected with rubber as an intermediate. We made a flange at both ends of the rubber body with the convex hull structures on both sides of the flange. The designed rubber flange fits the outer sleeve better. To a certain extent, the existence of the inner skeleton supports the rubber body and reduces the possible circular motion of the rubber body in the process of vehicle running. The outer ring in the middle of the rubber body was designed with rounded corners to form a gap between the rubber body and the outer sleeve, to reduce the influence of temperature deformation of the bushing and reduce the possible impact area. According to the above description and the principle of simulation, we can determine the flexible contact pairs: contact pair 1 is the contact between the upper flange surface of the rubber body and the steel plate (the steel plate was added to simulate the contact condition of the upper end of the bushing with other components), contact pair 2 is the contact between the lower flange surface of the rubber body and the outer sleeve, as shown in Figure 2d. In the contact property, we adopt the penalty friction formulation as tangential behavior; the friction coefficient is 0.5. Additionally, normal behavior is hard contact. In addition, we added the heat generation option to research the frictional heat generation phenomenon.

Taking into account the complexity of the overall model, the modeling process needs to be briefly described. First, we built the complete models of the rubber body, the outer sleeve and the steel plate in CATIA V5R21 software (Dassault Systemes, Paris, France), as shown in Figure 2a–c. Because only the ends of the bushing and sleeve are the object of study, we keep this part only after cutting the rubber body. In order to simplify the subsequent meshing process, the symmetrical rubber body and sleeve models were divided into 1/18 according to the angle of 20°. Then, we input the processed geometric models into HyperMesh 14.0 (Altair, Troy, MI, USA) to finish the meshing and circular arraying. Regarding the mesh properties, the rubber body and the metal parts would be set in ABAQUS to C3D8T and C3D8RT, respectively. Finally, the above components were assembled in ABAQUS, as shown in Figure 2d. To apply displacement excitation to the model, the rubber inner wall was coupled to a reference point RP_1_.

### 2.3. Material Mechanical Behavior of Rubber

**Figure 3:**
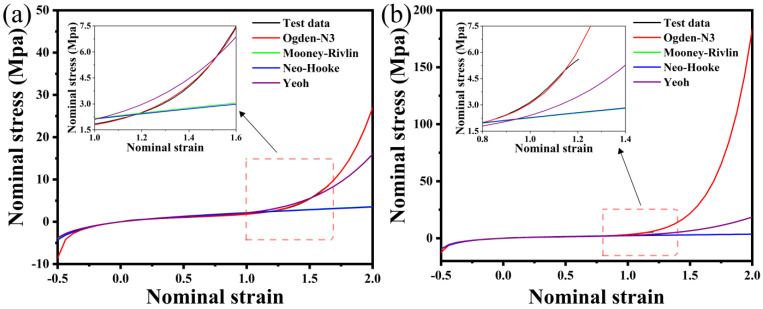
Fitting curve of the hyperelastic constitutive model: (**a**) uniaxial tensile; (**b**) plane tensile.

The material used in this study is NR70. As a hyperelastic material, it is necessary to select an appropriate hyperelastic constitutive accurate model and parameters in finite element simulation. We can use the fitting function of ABAQUS 2019 (Dassault Systemes, Paris, France) to process the experimental data. Firstly, two sets of data obtained from the tests (uniaxial and plane tensile tests) were imported into ABAQUS 2019, and the system output the stress–strain relation curve of the material and the parameters of each constitutive equation according to the least squares theory. Then, the curves of the four hyperelastic constitutive models were compared with the basic test curves, and the results are shown in Figure 3. It can be seen that in the initial stage, the effect of the Mooney–Rivlin and the Yeoh model is best. With the increase of strain, Ogden-N3 obviously fits more closely with the test curve. This indicates that the fitting curve of Ogden-N3 is the most consistent with the test curve in the overall range. Therefore, the Ogden-N3 constitutive model is selected as the hyperelastic model. Ogden is one of the constitutive models established based on the phenomenological method, and its constitutive relation is summarized by the expression of strain energy function as

(1) $$U=\sum_{i=1}^{N}\frac{2\mu_{i}}{\alpha_{i}^{2}}(\overset{-}{\lambda_{1}^{\alpha_{i}}}+\overset{-}{\lambda_{2}^{\alpha_{i}}}+\overset{-}{\lambda_{3}^{\alpha_{i}}}-3)+\sum_{i=1}^{N}\frac{1}{D_{i}}{\left(J-1\right)}^{2i}$$

where $\overset{-}{\lambda_{i}}$ refers to the principle stretches that satisfies the relationship $\overset{-}{\lambda_{1}}\overset{-}{\lambda_{2}}\overset{-}{\lambda_{3}}=1$ ; J is the volume ratio; and $\mu_{i}$ and $\alpha_{i}$ are dimensionless shearing coefficients that are obtained by fitting with the stress–strain curve of the material. The value of $D_{i}$ is 0 here because we assume that the material is incompressible. The Ogden-N3 parameters of rubber material are shown in Table 2.

It is well known that rubber bushing is an important part of suspension system for vibration isolation and shock mitigation, because it bears dynamic load excitation during vehicle operation and exhibits a certain frequency and amplitude dependence. In other words, the rubber bushing exhibits dynamic behavior. In this paper, Prony N3 is the viscoelastic constitutive model of rubber material according to the relevant data in the relevant database. The shear stress τ(t) of hyperelastic materials, such as rubber, during stress relaxation can be expressed as

(2) $$\tau \left(t\right)=G_{0}\left[\gamma -\int_{0}^{t}{\dot{g}}_{R}\left(s\right)\gamma \left(t-s\right)ds\right]$$

where $G_{0}$ is the dimensionless initial shear modulus that satisfies the relation $g\left(t\right)=G\left(t\right)/G_{0}$ . G(t) is expanded by the Prony series as

(3) $$g\left(t\right)=1-\sum_{i=1}^{N}g_{i}\left(1-e^{-t/\tau_{i}}\right)$$

where $g_{i}$ and $\tau_{i}$ denote, respectively, the dimensionless shear-relaxation modulus and relaxation time associated with the material. Parameters of the Prony N3 viscoelastic constitutive model are shown in Table 3. Moreover, the metal parts are set as non-deformable rigid bodies, which can also save computing resources at the same time.

## 3. Results and Discussion

The deformation analysis of rubber bushing is a complex nonlinear large deformation problem, which combines geometric nonlinearity, boundary condition nonlinearity and material nonlinearity. Therefore, two explicit analysis steps of dynamic thermal-displacement coupling were set to complete the quasi-static analysis of the rubber bushing in the Step module of ABAQUS. In the Initial Step, the rubber body, steel plate and outer sleeve are fixed. In Step-1, displacements of −1 mm and 1 mm are applied to the plate and outer sleeve to contact and compress the convex hull structure on the rubber body. In Step-2, we applied a radial displacement of 2.5 mm or an axial displacement of −3 mm to the RP_1_ coupled to the inner wall of the rubber body, referring to the working condition of the rubber bushing. Then, we set the ambient temperature to 20 in the Predefined Field, which is 20 °C.

### 3.1. Comparative Analysis of Radial Contact Equivalent Stress

**Figure 4:**
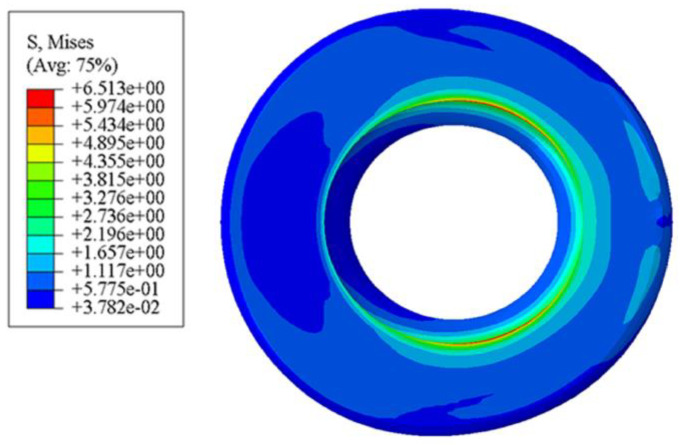
Contact equivalent stress diagram of standard rubber bushing.

**Figure 5:**
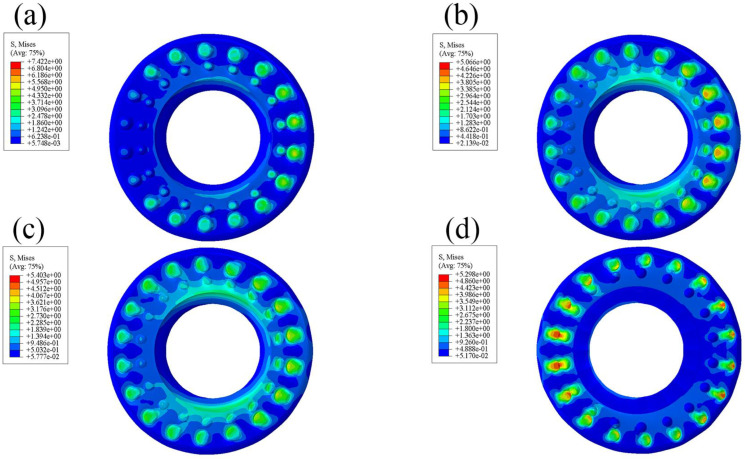
Contact equivalent stress diagram of bionic non-smooth surface: (**a**) type1; (**b**) type2; (**c**) type3; (**d**) type4.

From the contact equivalent stress diagram of the standard rubber bushing (Figure 4), it can be seen that there is an obvious stress concentration at the junction between the upper surface and the inner wall. When the rubber is subjected to external force, the equivalent stress at the innermost ring of the smooth surface is the largest, indicating that it can be easily damaged here. As can be seen from Figure 5, the maximum equivalent stress of the bionic non-smooth surface appears at the convex hulls, and the equivalent stress distribution presents rings that decrease gradually from the inside to the outside. Among them, the annular distribution effect of the type2 rubber model is the most obvious, followed by the type3 model. The annular distribution of the equivalent stress can effectively alleviate the problem of serious stress concentration on the rubber surface. The convex hulls can also absorb some stress with their own micro-strain, which can also reduce the possibility of rubber damage. In ABAQUS, since the size unit of the model is set to mm, the unit of stress and pressure is MPa. In addition, the equivalent stress values of the bionic-designed bushing are different, but they are close to the standard bushing’s value. Therefore, the equivalent stress of the bionic-designed bushing can meet the application requirements [31].

### 3.2. Comparative Analysis of Radial Contact Strain

**Figure 6:**
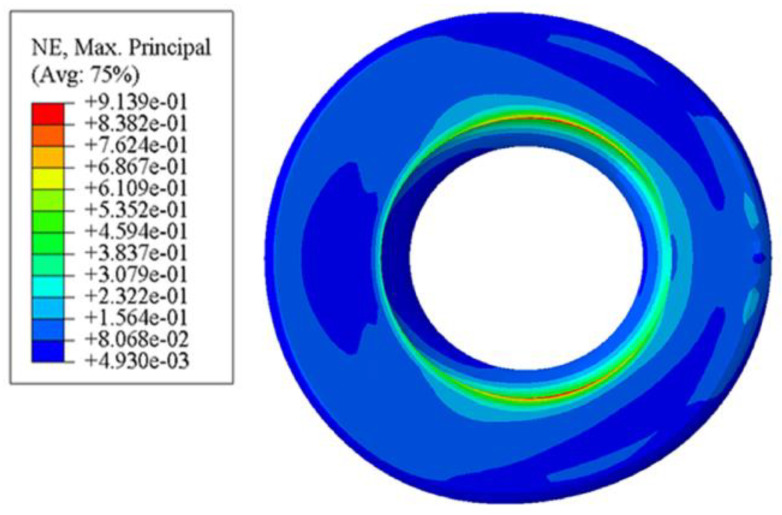
Contact strain diagram of standard rubber bushing.

**Figure 7:**
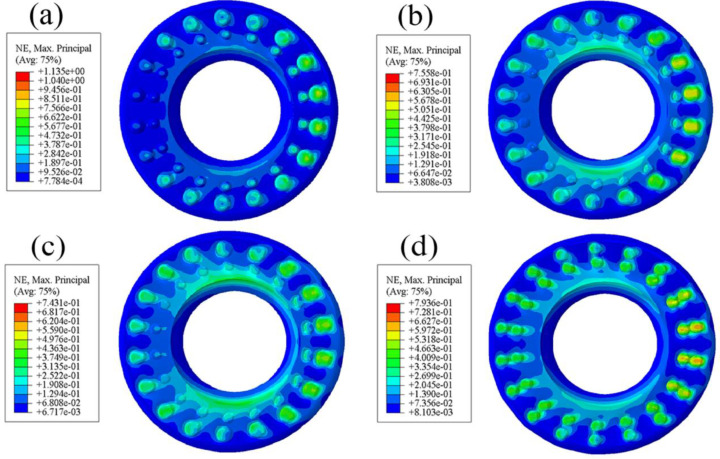
Contact strain diagram of bionic non-smooth surface: (**a**) type1; (**b**) type2; (**c**) type3; (**d**) type4.

The rubber bushing in this paper belongs to the inner sleeve bonding outer sleeve pressing type, that is, the inner wall of the rubber body and the inner sleeve are bonded together. It can be seen from Figure 6 that the strain and stress diagrams of the standard bushing exhibit similar characteristics: the strain in this paper is dimensionless and the maximum strain is also at the junction of the upper surface and the inner wall. When large strain occurs at the junction, the rubber is prone to cracking, resulting in a considerable safety hazard. It can be observed from Figure 7 that the deformation of the bushing is also roughly distributed in an annular shape that gradually decreases from the inside to the outside, and the maximum strain occurs at the convex hulls and their surroundings. This distribution reduces the likelihood of cracking.

### 3.3. Comparative Analysis of Radial Friction Heat

**Figure 8:**
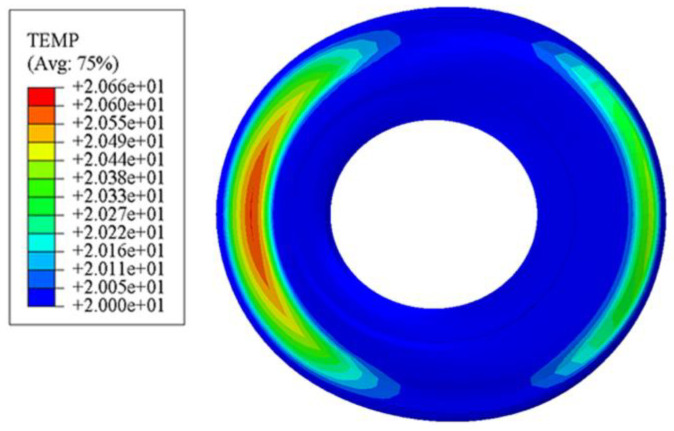
Friction heat diagram of standard rubber bushing.

**Figure 9:**
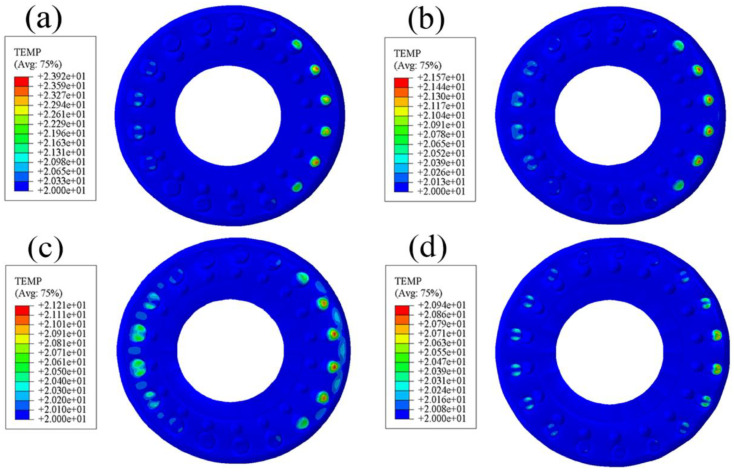
Friction heat diagram of bionic non-smooth surface; (**a**) type1; (**b**) type2; (**c**) type3; (**d**) type4.

Observing Figure 8, it can be found that there is a large red area with relatively high temperature on the surface. Only by observing the value of temperature can we see that the increase of the highest temperature is very small. However, the working state of the rubber bushing is dynamic as well as static, that is, the bushing surface may withstand a short time (0.02 s, 0.01 s, even less) and a high frequency (50 Hz, 100 Hz, or even higher) dynamic cyclic load. Therefore, in the process of relative motion between the smooth surface and the metal part, a large amount of friction heat may be generated. At the same time, because the contact area of the friction pair is large, the contact between the rubber and the outside air is quite insufficient, resulting in heat accumulation on the surface of the rubber, and the heat may be transferred to the interior in the form of heat transfer. However, in the process of driving the car, the movement of the bushing in multiple directions is short and rapid, and the relative movement time of the friction pair is short. A large amount of frictional heat cannot be transferred to the interior in such a short time, so it is mainly concentrated on the working surface. In view of heat accumulation on working surfaces being a major cause of thermal fatigue and wear, the smooth surface is more likely to be damaged. It can be observed from Figure 9 that the temperature of some convex hulls and their surrounding areas are relatively higher than the rest. The reason is that the existence of the bionic convex hull structures with an appropriate height can not only reduce the contact area between the non-smooth surface and the the metal part, but also increase the degree of contact between the friction pair and the air. Under the influence of the above two factors, the speed of heat convection between the non-smooth surface and the air is increased, which is conductive to the frictional heat dissipation of the whole friction pair. As a result, a large area of high temperature will not be generated. In the process of dry friction between the rubber body and the the metal part, the bionic convex hull structures alleviate the uneven temperature field caused by frictional heat generation to reduce the fatigue and wear of the rubber bushing in the working process.

### 3.4. Comparative Analysis of Radial Contact Pressure

**Figure 10:**
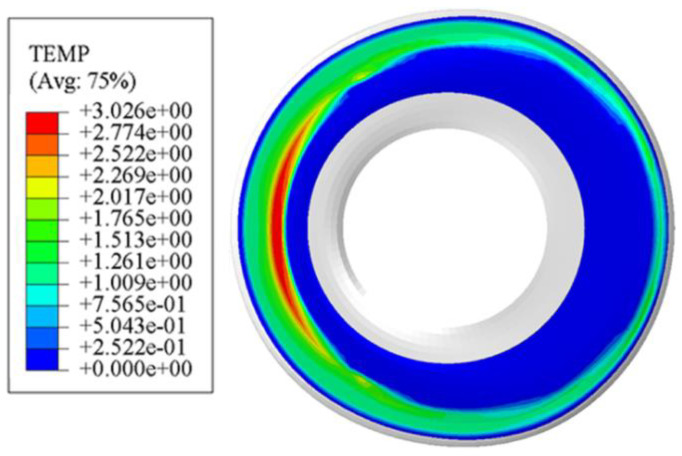
Contact pressure diagram of standard rubber bushing.

**Figure 11:**
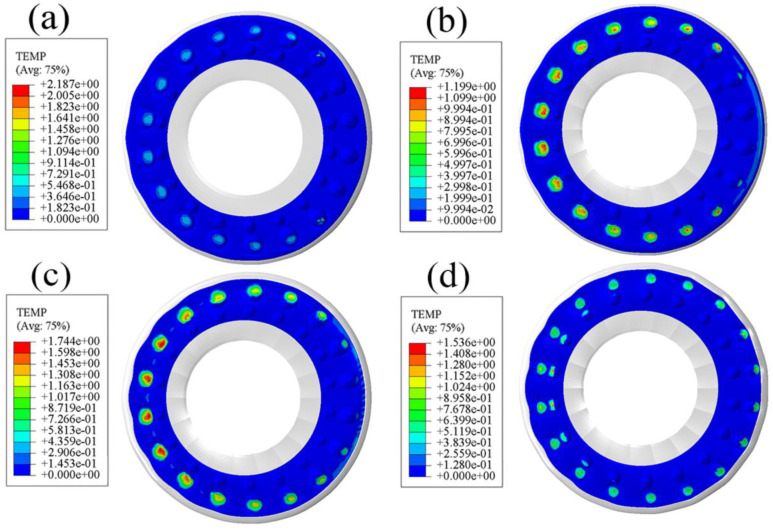
Contact pressure diagram of bionic non-smooth surface: (**a**) type1; (**b**) type2; (**c**) type3; (**d**) type4.

It can be seen from Figure 10 that there is continuous stress concentration on the smooth surface, and the location is almost coincident with the high temperature region of frictional heat. The stress concentration position is worn first, and a lot of frictional heat accumulates here; in turn, the working surface is worn faster. Observing Figure 11, it can be seen that the contact pressure of all bionic surfaces is in a relatively uniform point-like distribution state and the maximum value is much lower than the standard bushing, which is conducive to reducing the wear of the working surface.

### 3.5. Comparative Analysis of Axial Contact Equivalent Stress

**Figure 12:**
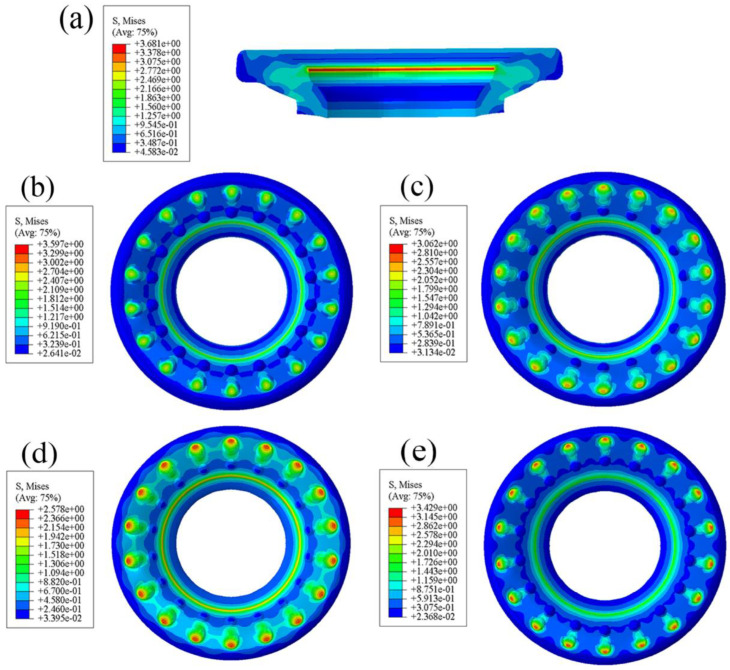
Diagram of contact equivalent stress: (**a**) section of standard bushing; (**b**) type1; (**c**) type2; (**d**) type3; (**e**) type4.

According to Figure 12, the equivalent stress maximum of the bionic surface is smaller than the smooth surface (3.681 MPa) and their positions are very different. It is obvious that there are partial high stress concentration areas at the top of the convex hulls, and there are obvious low stress areas around the bottom of the convex hulls. The maximum stress of the convex hull on the upper surface is 2.4, 2.4, 2.3, and 3.1 MPa. We can also observe that there is a ring-shaped stress gradient distribution on the smooth surface; the equivalent stress of the type3 and type4 surfaces is roughly distributed in a band-shape, and the equivalent stress of the type1 and type2 surfaces is distributed in a point-shape. The band-like and point-like distributions indicate that the stress transition is relatively gentle and the stress distribution on the bushing surface is relatively uniform. On the contrary, the existence of a continuous stress concentration distribution on the smooth surface makes it more prone to large-area wear than the bionic surface.

### 3.6. Comparative Analysis of Axial Contact Strain

**Figure 13:**
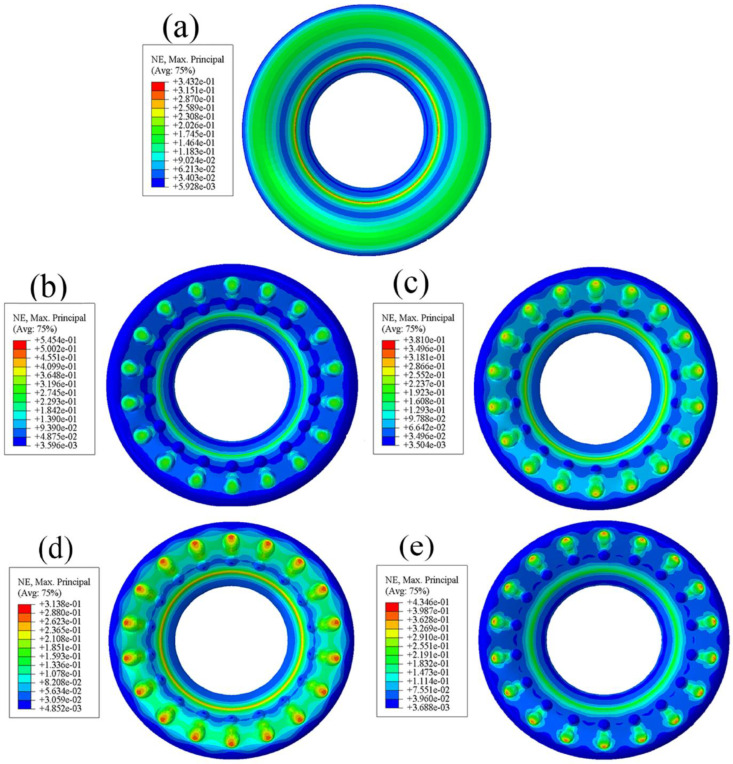
Diagram of contact strain: (**a**) type1; (**b**) type2; (**c**) type3; (**d**) type4; (**e**) standard bushing.

It can be seen from Figure 13 that the maximum strain of the bionic surface appears in the convex hulls and their vicinities. The maximum of the non-smooth surfaces, except for type3, is larger than that of the smooth surface. The contact surface whose stiffness is reduced by the convex hulls is more easily deformed when it comes into contact with the outer sleeve or steel plate, so that the surface generates more strain energy during friction. According to the law of conservation of energy, the increase of strain energy may reduce the frictional resistance effect of the friction pair during the working process, so that the effects of uniform and slow release stress are realized [32].

### 3.7. Comparative Analysis of Radial Friction

**Figure 14:**
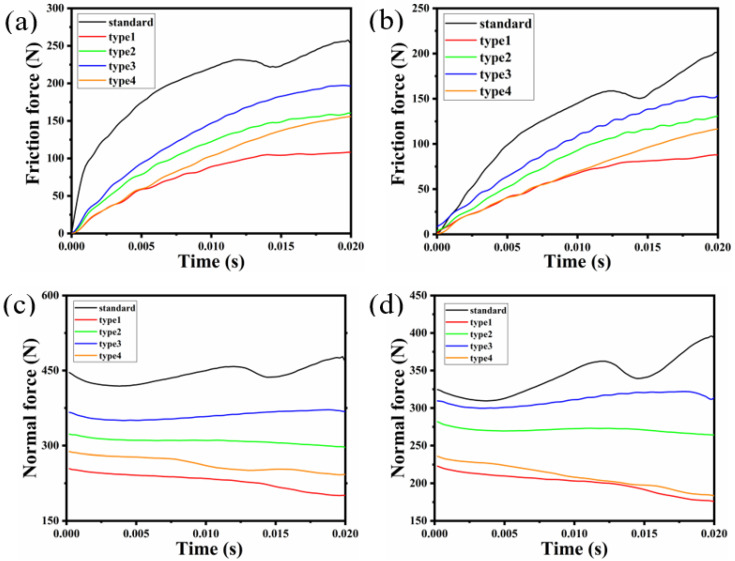
Contact friction force curve of (**a**) contact pair 1 and (**b**) contact pair 2. Contact normal force curve of (**c**) contact pair 1 and (**d**) contact pair 2.

It is observed from Figure 14 that the contact friction force on the surface of the standard rubber bushing is much greater than that of the rubber bushing with the bionic convex hull structure. Compared with the smooth surface, the contact friction force of the type1 model is the smallest, which is reduced by 57.17% and 56.93% in the radial and axial conditions, respectively. This shows that the existence of the convex hull morphology on the bionic non-smooth surface plays the role of drag reduction and wear resistance. In the initial stage of the relative motion of the friction pair, the contact areas of the type1 and type4 models are similar in size, so that the frictional force is very close at the beginning of the motion, and the curves of the contact frictional force are almost coincident. However, the contact area changes with the movement time, and the friction force curve of the type4 model develops to be slightly higher than that of the type1. The boundary condition in this simulation is displacement constraint, and the compression amount is constant. Therefore, none of the normal force curves in Figure 14 change dramatically.

## 4. Reliability Verification

The flexible convex hulls deform greatly and do not embed with the rigid body under the excitation of radial or axial displacement. At this time, there is mutual movement or mutual movement trend between the friction surfaces, and the equivalent friction coefficient between the contacting friction pairs is

(4) $$\mu_{eq}=\frac{F_{f}}{P}$$

where $F_{f}$ is the friction force and P is the normal force.

**Figure 15:**
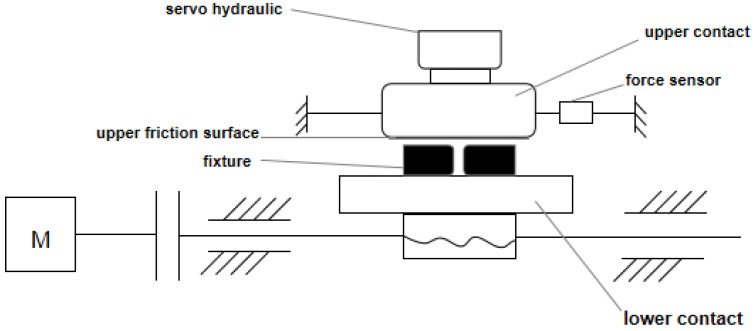
Scheme of the test system (M is for motor).

The scheme of the test system is shown in Figure 15. The sheet metal is bolted to the lower surface of the upper contact body and the sheet is the upper friction surface. The normal force is applied to the upper contact body by the servo hydraulic press, so the normal force is controllable. The fixture on the lower contact body is used to fix the rubber bushing. The motor drives the lead screw to rotate, and the lower contact body moves accordingly. At this time, the upper and lower surfaces move relative to each other, resulting in friction. The force sensor is connected with the upper contact body, and tension values are displayed and recorded on the computer in real time. This tension is $F_{f}$ . Since it is difficult to measure the friction coefficient at high speed, the lower contact body is reciprocated at a speed of 6 cm/s during the experiment. Finally, the equivalent friction coefficient can be calculated by Equation (4). The results of the maximum equivalent friction coefficients are shown in Table 4.

**Figure 16:**
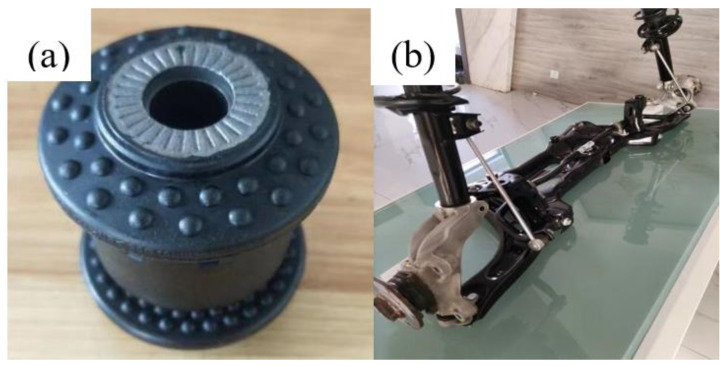
(**a**) Bionic rubber bushing. (**b**) Automobile front axle rocker arm.

There are two possible reasons for the difference between the values of the simulation and the experimental friction coefficients: (1) The inescapable errors in the test disable the test process from reaching the ideal state, such as the resistance of the test system, so the test speed cannot reach the simulation speed; (2) The dynamic characteristics are more closely related to the constitutive relationship. However, due to the lack of creep test and relaxation test conditions of rubber materials, the accuracy of the viscoelastic constitutive model is not high enough. In general, the average relative error between simulation and test results is 12.3%, which is still within the acceptable range. Hence, the simulation results can be considered accurate. It is obvious that the test value of type2, the simulated value and the test value of type4 are all greater than 0.6, indicating that incomplete relative sliding has occurred between the friction pairs. The maximum friction coefficients of type1 and type3 models are smaller than standard bushing, indicating that the existence of convex hulls with different sizes of the inner and outer rings has the effect of reducing the friction coefficient. The friction coefficient of the type4 model is the biggest, which is even about 29.6% higher than the standard bushing. In view of this phenomenon, we conclude that the reasons for this are that the average radius of convex hulls on the type4 surface is largest, but the height is smallest. This means that the convex hulls on the type4 surface was squashed more to be flatter. The contact area between the rubber body and the metal part includes not only the surface of the convex hulls, but also the plane area. Under the radial force, the distance between convex hulls in the X direction decreases. In addition, from the contact equivalent stress diagram and friction heat diagram, it can be seen that the stress concentration area and high temperature area of the convex hulls on the surface of type4 are relatively large. Similarly, the phenomenon also occurs in the test. Under the influence of heat and stress, the surface of the convex hull softens during the test, so the hardness decreases, and the actual contact area increases. Under the influence of the above factors, the actual contact area of the friction pair increases, and the equivalent friction coefficient increases correspondingly. As a result, the equivalent friction coefficient of type4 is greater than the standard bushings. To sum up, the type1 rubber bushing model (Figure 16) is the bionic rubber bushing model exhibiting the best drag reduction and wear resistance.

## 5. Conclusions

The convex hull of the dung beetle head’s surface is used as a prototype for bionic design, and we design a rubber bushing with representative microstructures on the surface based on bionic principles. The real irregular convex hull structure of the dung beetle head’s surface is simplified reasonably according to the principle of bionics and the necessary structural design parameters are determined. The tensile test data of the rubber material are fitted in the hyperelastic material property module that comes with ABAQUS. According to the fitting effect, Ogden N3-Prony N3 is selected as the hyper-viscoelastic constitutive model, and the parameters of constitutive model are also output. Both numerical simulation and experiment are adopted to establish a standard bushing model and four bionic bushing models, and then the mechanical properties of the bionic flexible friction pair are systematically analyzed from stress, strain, thermal effects and other aspects. The conclusions of this paper are summarized as follows:

1. The bionic convex hull structure effectively relieves the stress concentration at the boundary of the rubber bushing, and the micro-strain of the bushing absorbs part of the stress to reduce the possibility of rubber cracking.
2. The convex hull structure increases the speed of heat convection and heat dissipation between the working surface and the air, and relieves wear caused by an uneven temperature field.
3. The friction coefficient of the contact surface of the type4 model is the largest, indicating that convex hulls with a very small height may increase the actual contact area between contact pairs and, thus, increase the friction coefficient. The friction force of the contact surface of the type1 model is the smallest, indicating that convex hulls of different sizes can significantly reduce the friction force of the rubber contact surface. On the premise that the conditions of use are met, the comprehensive drag reduction and wear resistance effect of type1 bionic rubber bushing is the best.
4. In this paper, the front axle rocker arm bushing designed based on the principle of bionics can reduce abnormal noise during the driving process of the car, and provide theoretical guidance for the design and development of the bushing.

## Figures and Tables

**Table 1:** Dimensions of the convex hull section.

Shape (mm)	R_1_	R_2_	H_1_	H_2_
type1	1.25	1.75	1.25	1.75
type2	1.25	1.25	1.25	1.25
type3	1.25	1.75	0.75	1.25
type4	1.25	1.75	0.5	1

**Table 2:** Parameters of the Ogden N3 hyperelastic constitutive model.

Constitutive Model	*μ_i_*	*α_i_*
Ogden N3	1.20182023	0.550907592
	1.680490803 × 10^−3^	11.3337265
	1.17984475 × 10^−3^	−13.4689097

**Table 3:** Parameters of the Prony N3 viscoelastic constitutive model.

Constitutive Model	*g_i_*	k_i_	τ_i_
Prony N3	0.1954	0.0401	0.5373
	0.14	0.0035	0.019
	0.0604	0.004	0.000203

**Table 4:** Maximum friction coefficient.

Shape	Simulation Data	Test Data	Error
standard bushing	0.512	0.541	5.664%
type1	0.499	0.539	8.016%
type2	0.496	0.607	22.379%
type3	0.490	0.534	8.980%
type4	0.664	0.776	16.867%

## Data Availability

The datasets used or analysed during the current study are available from the corresponding author on reasonable request.
